# Internal Herniation Causing Double Obstruction of the Small Bowel and Urinary Tract: A Rare Case Presentation

**DOI:** 10.7759/cureus.65483

**Published:** 2024-07-27

**Authors:** Athanasios Permekerlis, Eirini Gemousakaki, Christos Tepelidis, Panagiotis Fotiadis

**Affiliations:** 1 2nd Surgical Department, 424 General Military Hospital, Thessaloniki, GRC

**Keywords:** internal hernia beneath ureter, ureter reimplantation, ureter injury, internal hernia, urinary tract obstruction, small bowel obstruction

## Abstract

Small bowel obstruction is one of the most common urgent surgical conditions, caused by a variety of factors, with adhesions, malignancies, and hernias, internal and external, being the most common. Many types of internal hernias have been described in the literature; however, internal hernia caused by the ureter as a secondary complication of ureteroplasty is rare and only a few cases have been reported worldwide. This presentation discusses an interesting case of small bowel obstruction accompanied by obstruction of the urinary tract due to an internal hernia caused by the ureter. A 58-year-old female presented to the emergency department (ED) with acute pain in the abdominal and right lumbar region. Her surgical history includes hysterectomy, right ureter injury, and ureteroplasty performed 10 years ago. Clinical examination showed tenderness in the lower abdomen, positive Giordano's sign on the right, and metallic bowel sounds. A computer tomography scan revealed right-sided hydronephrosis, absence of excretion in the right urinary tract, and dilated loops of the small intestine. An exploratory laparoscopy revealed a small bowel loop strangulated by the ureter, followed by laparotomy, resection of a segment of the ileum, oblique anastomosis, and reimplantation of the right ureter. The patient was discharged eight days postoperatively without any complications. This case underscores the significance of surgical history in order to recognize even rarer causes of small bowel obstruction.

## Introduction

Internal hernia is the protrusion of abdominal contents, mostly small bowel loops, through a congenital or acquired defect in the peritoneum or the mesentery of the abdominal cavity. Several types of internal hernia have been described in the literature, having an overall incidence of less than 1%. Although they represent up to 5.8% of the total cases of small bowel obstructions, they are associated with a significant mortality rate. The most common types of internal hernia include paraduodenal (53%), pericecal (13%), foramen of Winslow (8%), transmesenteric and transmesocolic (8%), intersigmoid (6%), and retroanastomotic (5%) hernias [1].

In recent years, the performance of new surgical procedures, such as liver transplantation [2] and gastric bypass for bariatric treatment [3], has led to an increased incidence of internal hernias and the emergence of new types. In addition to clinical findings, radiographic findings are highly useful in the diagnostic approach to these cases, with an emphasis placed on the CT features [1].

Retro-ureteral small bowel incarceration is a rare type of internal hernia and only a few cases have been described in literature. These cases refer to patients who have undergone radical cystectomy, gynecological procedures, and ureter reimplantation or kidney transplantation [4].

We report a case of small bowel obstruction secondary to internal hernia beneath the ureter. The herniation led to double obstruction of the small bowel and urinary tract.

This study was previously presented as an ePoster at the 46th European Hernia Society (EHS) Annual Conference on May 29-31, 2024.

## Case presentation

A 58-year-old female presented to the emergency department with pain in the acute abdominal and right lumbar region, which had been ongoing for the past 8 hours, accompanied by nausea. The patient reported a surgical history of hysterectomy, right ureter injury and reimplantation 10 years ago (no more details regarding the type of surgery were available) and no history of any other medical conditions.

The patient appeared well-nourished, in good general condition, and her vital signs showed mild tachycardia. Clinical examination revealed tenderness in the lower abdomen, positive Giordano's sign on the right, and metallic bowel sounds. Laboratory tests showed a slight elevation of the inflammatory status, white blood cell (WBC) count of 12.78 k/μL (normal range: 4.5-11 k/μL), and C-reactive protein (CRP) level of 0.8 mg/dl (normal value <0.5 mg/dl). Serum electrolytes, urea, creatinine, and liver function tests were all within normal limits.

**Figure 1:**
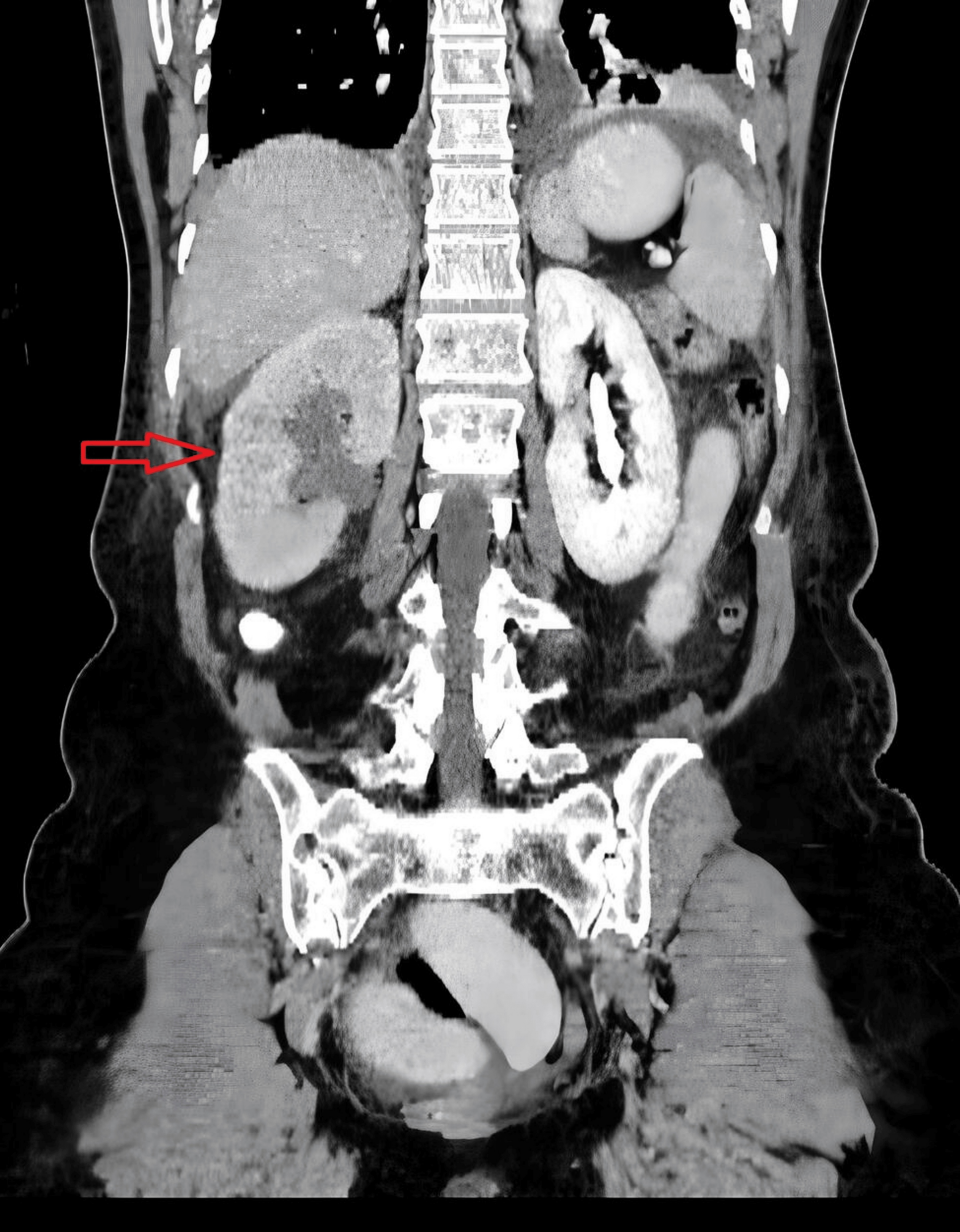
CT showing right-sided hydronephrosis with perinephric stranding and absence of excretion in the right urinary tract (red arrow)

**Figure 2:**
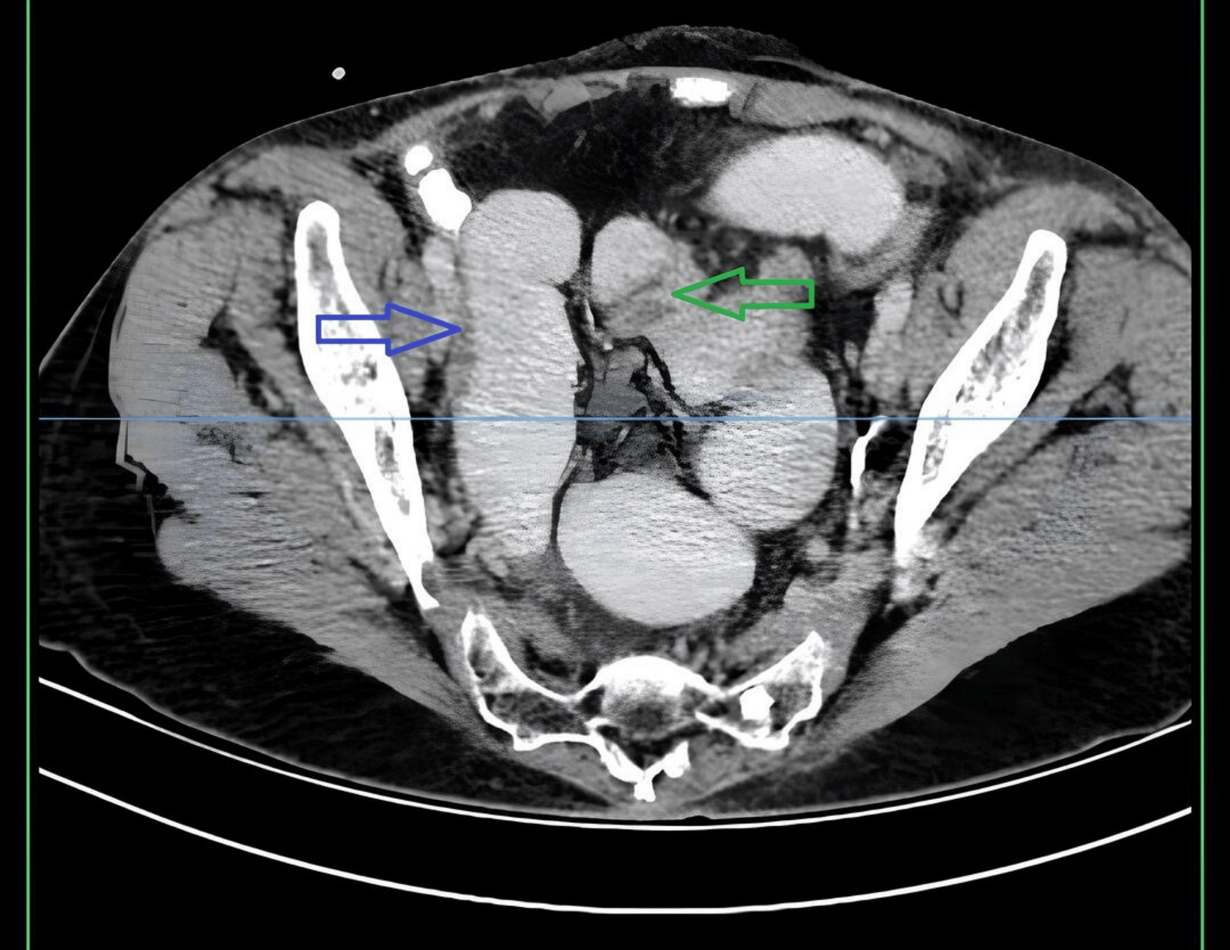
CT showing dilated loops of the small intestine in the pelvic cavity (blue arrow) and point of luminal narrowing of the right distal ureter (green arrow)

An abdominal X-ray was performed in an upright position, revealing dilated loops of small bowel and multiple air-fluid levels. An abdominal computed tomography (CT) scan with intravenous contrast showed right-sided hydronephrosis with perinephric stranding and absence of excretion in the right urinary tract (Figure 1, red arrow). In the pelvic cavity, dilated loops of the small intestine were observed (Figure 2, blue arrow), and at the same level, a point of luminal narrowing of the right distal ureter (Figure 2, green arrow). 

These findings were suggestive of the diagnosis of double obstruction in the small bowel and urinary tract. After obtaining the patient's informed consent, she underwent a diagnostic laparoscopy. Adhesiolysis was performed and the entrapped small bowel loop was identified with ischemic changes beneath the intraperitoneal right ureter. The surgery was converted to exploratory laparotomy. Resection of the strangulated segment of the ileum was performed and bowel continuity was established with a stapled side-to-side anastomosis. Following this procedure, the right ureter was retroperitonealized, reimplanted into the bladder, and a ureteral catheter was placed. The postoperative course was uneventful and the patient was discharged eight days postoperatively, with the ureteral catheter removed 30 days later. There were no surgical complications in the 12 months of follow-up.

## Discussion

Internal herniation beneath the ureter, with retro-ureteral small bowel incarceration and simultaneous urinary tract obstruction is a rare phenomenon and the literature contains very few cases of this double obstruction. The first instance was described by D.J. Hay in 1981 [5]. Since then, it has been reported at least 10 more times, and it seems to be occurring in patients who have a specific past surgical history. This history refers to intraperitoneal kidney transplantation, primary urological surgery, and gynecological procedures accompanied by ureteric reimplantation.

The preoperative diagnosis of an internal hernia is challenging and the clinical presentation is nonspecific and can vary. Without a heightened awareness, they can often be misdiagnosed. The patient's surgical history should be strongly considered in the diagnostic approach and rarer causes of gastrointestinal obstruction should be included in the differential diagnosis, based on each patient’s history. For example, Petersen's hernia occurs after Roux-en-Y gastric bypass [6]. Apart from clinical suspicion, abdominal CT is rapidly becoming the first-line imaging technique in these patients. CT can detect small bowel obstruction with high sensitivity and specificity and, in most cases, has the potential to identify an internal hernia as the cause [1]. In our patient’s case, we preoperatively suspected an internal hernia associated with the ureter based on a combination of the patient’s surgical history, physical examination, and findings from imaging.

Increased clinical suspicion will help intraoperative recognition of the ureter, as believing it to be a band adhesion and excision has been described. A case report published by Tovmassian et al. [7] outlines a case of internal herniation with an obstructed small bowel loop, beneath a transplant ureter in a patient who underwent renal transplantation. Intraoperatively, the transplant ureter had been mistaken for a band adhesion and was completely transected. Finally, the divided transplant ureter was reimplanted into the bladder in a repeat laparotomy, a complex procedure. In some cases, the placement of a ureteral catheter is useful to identify the ureter.

In order to prevent this type of internal hernia, retroperitonealization of the ureter in patients undergoing pelvic operations involving ureteral mobilization is significant. Intraperitoneal approach in kidney transplant is associated with a higher incidence of postoperative gastrointestinal complications including internal herniation beneath the ureter [8]. Surgical techniques are preformed to obliterate the sub-ureteric space to prevent further complications [9].

## Conclusions

The incidence of acquired internal hernias has increased in recent years due to the performance of new surgical procedures. High clinical suspicion in combination with the imaging findings will help the preoperative diagnosis, in order to avoid complications in the operating field. We experienced a patient with small bowel obstruction accompanied by obstruction of the urinary tract, due to an internal hernia caused by the ureter, as a secondary complication of ureter injury and reimplantation during a gynecological procedure.
